# Geospatial Analysis and Molecular Epidemiologic Study of Emerging Pulmonary Lophomoniasis in Iran: A National Registry-Based Study

**DOI:** 10.1155/2023/1039186

**Published:** 2023-06-07

**Authors:** Mohammad Amin Ghatee, Maryam Nakhaei, Ali Sharifpour, Mahdi Fakhar, Niloufar Mohamadi, Mostafa Soleymani, Siavash Abedi, Masoud Aliyali, Hossein Mehravaran

**Affiliations:** ^1^Department of Microbiology, School of Medicine, Yasuj University of Medical Sciences, Yasuj, Iran; ^2^Cellular and Molecular Research Center, Yasuj University of Medical Sciences, Yasuj, Iran; ^3^Iranian National Registry Center for Lophomoniasis, Imam Khomeini Hospital, Mazandaran University of Medical Sciences, Sari, Iran; ^4^Student Research Committee, Yasuj University of Medical Sciences, Yasuj, Iran

## Abstract

**Introduction:**

Bronchopulmonary lophomoniasis (BPL) is a protozoan pulmonary disease that has been reported sporadically, but its incidence has been increasing. However, the epidemiology and risk factors of the disease have not been clearly identified. The current study aims to identify BPL cases molecularly and assess the demographic and some environmental factors for the first time on the prevalence of BPL as a national registry-based study in Iran. *Methodology*. The study tested 960 patients with lower respiratory tract symptoms whose bronchoalveolar lavage samples were submitted from seven provinces of Iran to the Iranian National Registry Center for Lophomoniasis. They were tested for BPL by a newly developed polymerase chain reaction test. The study assessed the association of Normalized difference vegetation index (NDVI), digital elevation model (DEM), and geographic latitude as environmental factors and sex and age as demographic factors on the prevalence of BPL. Geospatial information systems methods and chi-squared and Pearson's correlation tests were used for the assessment of geographical and environmental factor effects and statistical analysis, respectively.

**Results:**

Of the 960 patients, 218 (22.7%) tested positive for BPL; the highest and lowest prevalence rates were reported from the south and northeast of Iran, respectively. The study found a correlation between geographic latitude and age with BPL prevalence, but no association was found for gender, NDVI, or DEM. Most patients were over 40 years old, and the rate of disease was higher in southern latitudes.

**Conclusion:**

Age and geographical latitude were found to be risk factors for BPL. More exposure to dust and/or chronic pulmonary problems may explain the higher prevalence of the disease in older adults. Higher rates of BPL in lower latitudes may be due to warmer weather and longer days, which can confine individual activities indoors and result in more contact with domestic insects and infected dust.

## 1. Introduction

Bronchopulmonary lophomoniasis (BPL) is primarily caused by the protozoan parasite *Lophomonas blattarum* (*L. blattarum*) [1]. The Lophomonadidae family is a member of the parabasalids (Metamonada phylum), a group of multiflagellated protozoa with a parabasal apparatus and a hydrogenosome similar to Trichomonadidae [2]. Lophomoniasis has been reported in both healthy immunocompetent and immunocompromised individuals, including those with a history of transplant, acquired immune deficiency syndrome, asthma, and allergic rhinitis around the world [3–5]. Data from a systematic review showed that lophomoniasis cases were reported from 10 countries, mostly from Asian countries, such as Iran and China. However, the global true burden of the disease is still unknown [6]. The main symptoms of BPL are cough, low-grade fever, and dyspnea. Prominent signs of BPL include lymphocyte reduction, increased neutrophilia, and eosinophilia [7]. Radiography may show patchy local shadowing, small diffuse and bilateral infiltrates, reticular infiltration, and lobular consolidation [8–10]. While almost all symptomatic infected cases have had lower respiratory tract (LRT) symptoms, rare cases of upper respiratory tract involvement, such as sinusitis, have also been reported [11, 12]. BPL is a rare infection of the respiratory system of humans [13, 14], mainly reported in patients with pulmonary problems who are resistant to antibacterial therapy [15, 16]. Infected dust with *Lophomonas* cysts, close contact with cockroaches and termites as invertebrate hosts, moisture, pulmonary problems, and underlying diseases are assumed to be disease risk factors [17, 18].

Microscopic examination is a routine method for the diagnosis of BPL. *Lophomonas* is a round-ovoid to pyriform protozoa with dimensions of 20–60 in 12–20 *μ*m, with a tuft of flagellates in a pole, longer flagellates in the center, and smaller ones in the circumference, and a round nucleus. However, this organism is frequently misdiagnosed as pulmonary nasal epithelial cells [19]. Fakhar et al. developed a polymerase chain reaction (PCR) approach based on the diagnosis of a 214 bp fragment of small subunit ribosomal RNA (SSU rRNA) that can be used for a reliable diagnosis and confirmation of suspicious cases [20].

Geographic information systems (GIS) are computer-based approaches that have been used to assess the effect of different factors, including geo-climatic determinants on the prevalence and distribution of different infectious diseases, especially vector-borne parasitic diseases [21]. This technology uses data from satellites and earth bases to determine risk factors and hazard zones and generate prediction models of infectious disease expansion that help control public health problems [22].

Although the transmission route of *Lophomonas* to humans is unclear, some scientists believe that BPL has been transmitted by inhalation or ingesting *L. blattarum* cysts excreted from the hindgut of cockroaches, termites, and mites [4]. Therefore, environmental factors may play a role in the prevalence and transmission rate of BPL between hosts and to humans regarding the probable vector or airborne nature of diseases. Some studies hypothesized the effect of some geo-climatic factors on the prevalence of BPL, but to the best of our knowledge, no geographical information system-based study demonstrated disease distribution and analyzed environmental factors on the occurrence of BPL. This study is a comprehensive study of BPL in the world, including 960 cases from seven provinces of Iran. Therefore, we developed a large-scale GIS-based study to investigate the association of some demographic and environmental factors with BPL in Iran.

## 2. Methodology

### 2.1. Patient Data and Study Areas

In this descriptive analytical cross-sectional study, data from 1001 patients with LRT symptoms and their bronchoalveolar lavage (BAL) samples were submitted to Iranian National Registry Center for Lophomoniasis for molecular diagnosis of BPL at Mazandaran University of Medical Sciences, Sari, Mazandaran Province, Northern Iran, between 2018 and 2021. Demographic data, including age, gender, and province of residence, were obtained from patients' diagnosis requisitions that were sent for samples. Patients primarily came from seven provinces of Iran, including Tehran (Center), Mazandaran, Golestan (North), Khorasan Razavi (Northeast), Ardabil (Northwest), Chaharmahal and Bakhtiari (West), and Kerman (Southeast). There were low numbers of cases from some other provinces as well as Afghanistan and Azerbaijan, which were omitted. Finally, 960 cases from the aforementioned seven provinces were included in the present study. The study was approved by the Ethical Committee of Mazandaran University of Medical Sciences (ethical code: IR.MAZUMS.REC. 1397.2969).

### 2.2. Molecular Diagnosis

#### 2.2.1. DNA Extraction

A BAL sample (200 *μ*L) was subjected to 200 *μ*L of lysis buffer consisting of 50 mM Tris–HCl (pH 7.6), 1 mM Ethylenediaminetetraacetic acid, and 1% Tween 20 and homogenized. The samples were incubated overnight at 45°C with 20 *μ*L of proteinase K solution (20 mg enzyme/mL). After strong shaking, 200 *μ*L of phenol : chloroform : isoamyl alcohol (25 : 24 : 1) solution was added, and the samples were centrifuged at 14,000*g* for 15 minutes. The supernatant was collected and transferred to a new microtube, where 400 *μ*L of absolute ethanol was added and kept at −20°C for 2 hours before centrifugation at 14,000*g* for 15 minutes. Then, 200 *μ*L of 70% ethanol was added to the precipitate and centrifuged, and the precipitate was suspended in 50 *μ*L of double-distilled water and stored at 4°C until use.

### 2.3. Conventional PCR

A 214-bp DNA fragment was amplified to detect *Lophomonas* infection using forward (F) 5′-GAG AAG GCG CCT GAG AGA T-3′ and reverse (R) 5′-ATG GGA GCA AAC TCG CAG A-3′ primers for small SSU rRNA. Each reaction volume consisted of 12.5 *μ*L of the Master Mix (Fermentas, Inc.), 1 *μ*L of each primer, 5 *μ*L of the extracted DNA, and 5.5 *μ*L of distilled water. The cycling program included 35 cycles performed in a thermocycler (Corbett Research, Sydney, Australia), with an initial denaturation at 94°C for 2 minutes, followed by 40 cycles of 94°C for 1 minute, 57°C for 1 minute, and 72°C for 1 minute, and then a final extension at 72°C for 3 minutes. PCR products were electrophoresed on a 1.5% (w/v) agarose gel and visualized by UV transillumination after staining with SafeView TM DNA Stains (Applied Biological Materials, Inc.).

### 2.4. Geospatial and Remote Sensing Data and Analysis

The digital elevation model (DEM) layers of the selected provinces were extracted from Iran DEM using the clip gadget of a raster processing tool. Normalized difference vegetation index (NDVI) images for the studied areas and period were obtained from the United States Geological Survey Earth Explorer website (https://earthexplorer.usgs.gov/). The images were MOD 13Q1 satellite products captured every 16 days at 250-m spatial resolution from the Moderate Resolution Imaging Spectroradiometer Vegetation Indices of NASA LPDAAC collections. NDVI ranges between −1 and 1, with negative values indicating water, values close to 0 indicating bare soil, and positive values indicating denser vegetation. Iran is covered by five NDVI image sections for each time. The average pixel value of all images (raster) of each section was calculated by the raster calculator tool, and the scale was set to 0.0001 to obtain a single NDVI raster layer for each section. These sections were then unified by mosaic to a new raster tool, and each provincial NDVI layer was generated by clipping it based on the vector polygon of the corresponding province. The mean pixel value of the raster layer was calculated by zonal statistics as shown in Table 1 for each province. All analyses were performed using ArcGIS 10.5 (ESRI, Redlands, CA, USA).

### 2.5. Statistical Analysis

Descriptive data were presented as mean and standard deviation, frequency, and percentage. The relationship between variables was examined using chi-squared and Pearson's correlation tests.

## 3. Results

### 3.1. Demographic and Conventional PCR Results

The study included 960 patients with LRT symptoms, of whom the majority were male (68.1%). The mean age of the patients was 52.77 years, and the standard deviation was 20.69. The age range was 1–95 years, with most patients (41.4%) being over 60 years old. Of the LRT symptomatic patients, 218 (22.7%) tested positive for BPL by PCR. Among the confirmed BPL patients, 61.9% were male, and the mean age and standard deviation were 56.45 and 19.54, respectively. The age range was 2–95 years, with the highest percentage of confirmed patients (48.6%) being over 60 years old, and the lowest percentage (1.7%) being in the 1- to10-year age group. No significant association was found between PCR results and gender (*p* = 0.993), but a significant association was found for age (*p* = 0.027). Age and gender data were available for 735 and 958 cases, respectively, based on data received from referring centers (Table 1). Since demographic data, including age and gender, were not available for all received samples, statistical analysis for the association of PCR results with age and gender was limited to these 735 and 958 cases, respectively.

**Figure 1:**
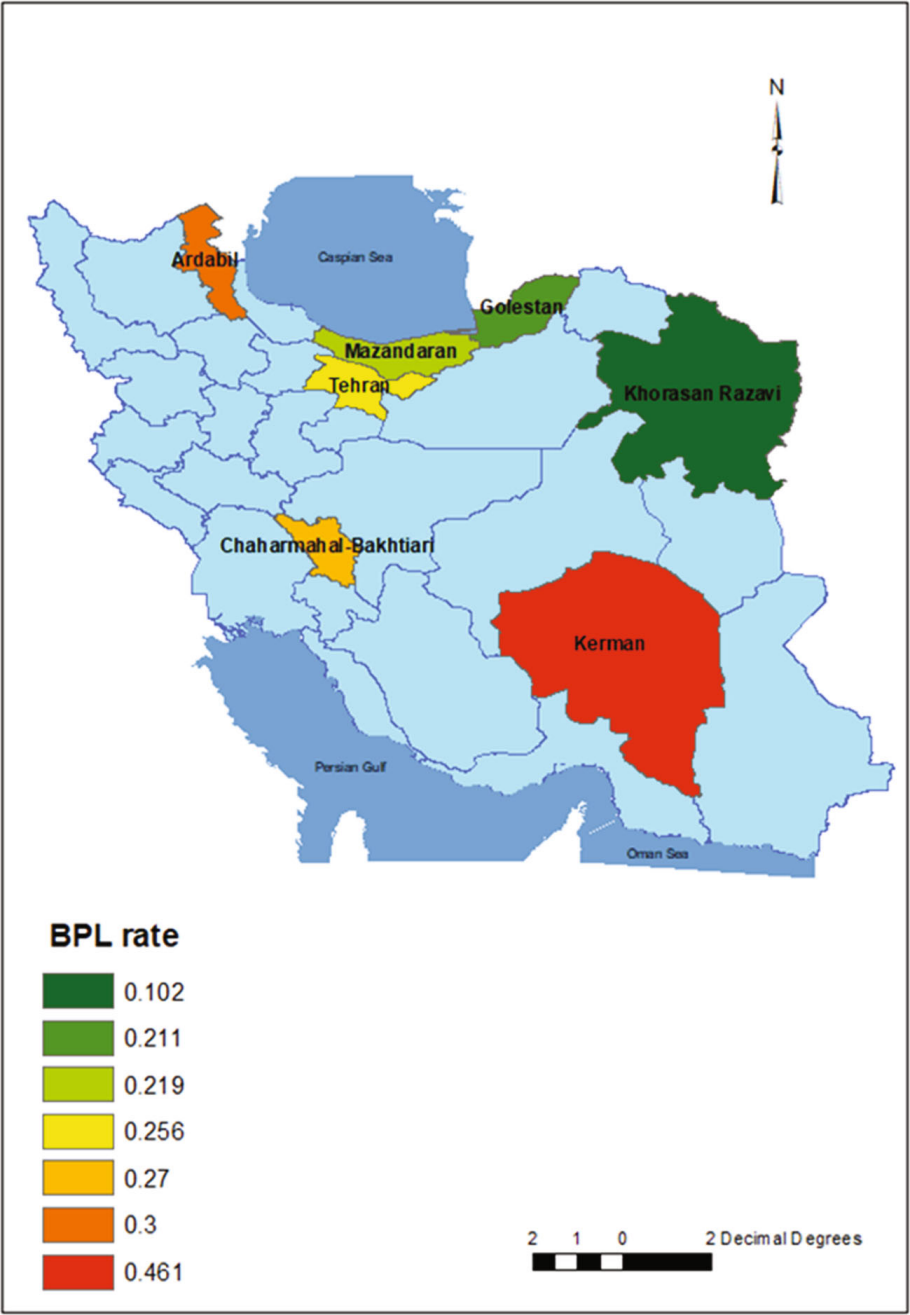
Distribution of BPL rate in studied provinces.

The highest rate of PCR positivity was found among patients from Kerman province, followed by Ardabil, Chaharmahal and Bakhtiari, Tehran, Mazandaran, Golestan, and Khorasan Razavi, respectively. There was no significant association between the frequency of patients referred from different provinces and PCR results (*p* = 0.093; Table 1; Figure 1).

### 3.2. Geographical Analysis

**Figure 2:**
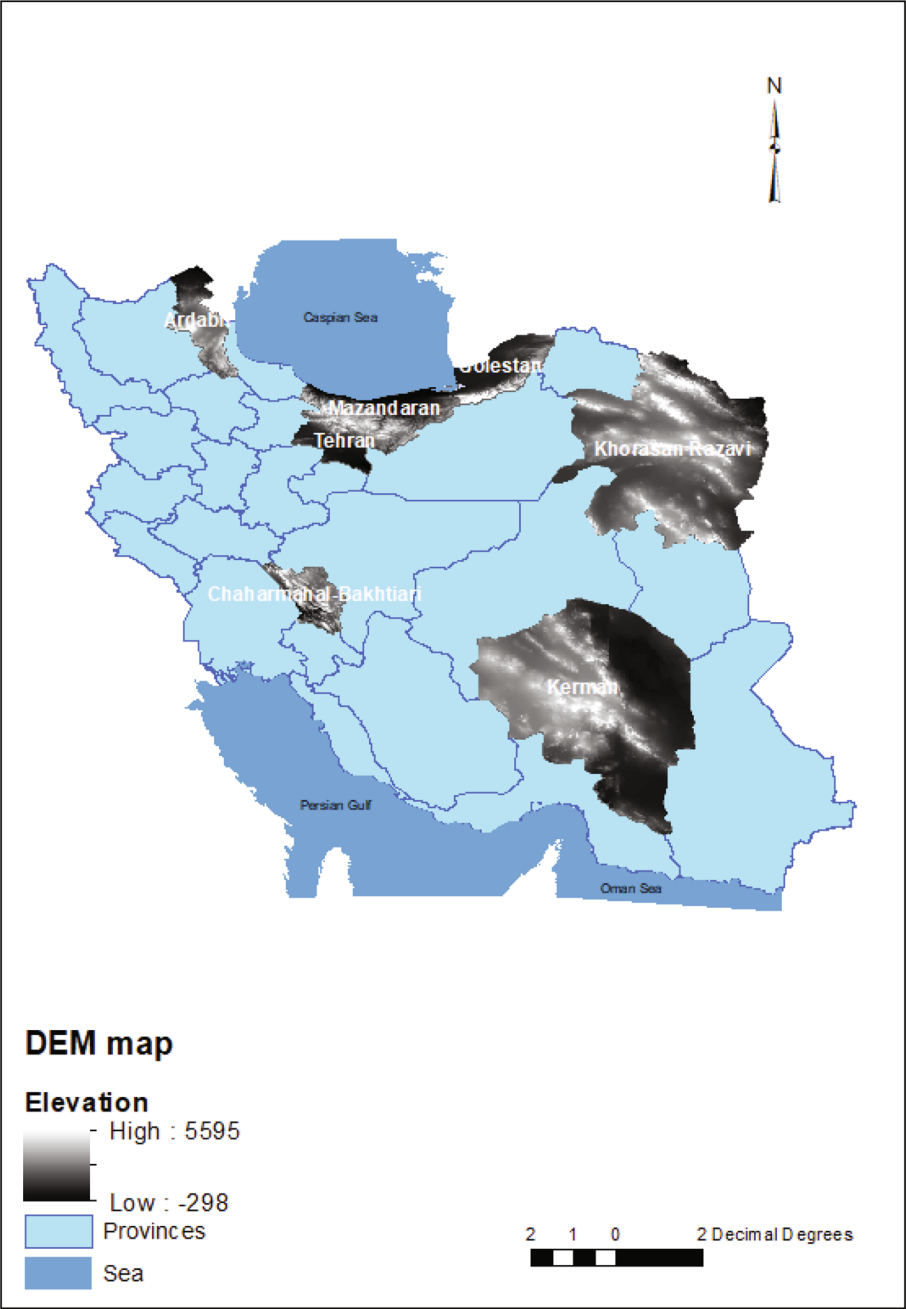
DEM map of studied provinces.

**Figure 3:**
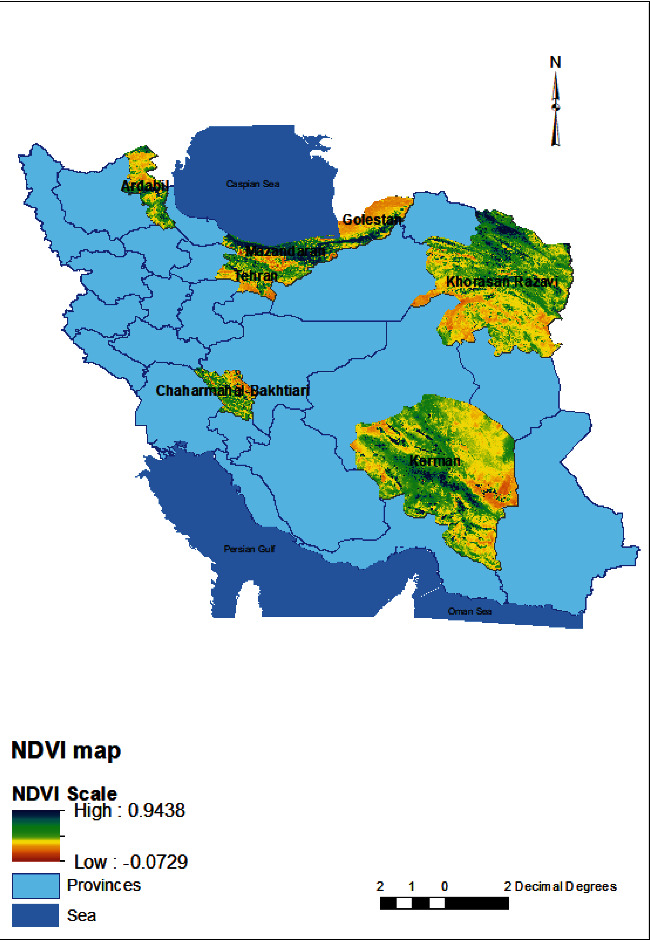
NDVI map of studied provinces.

Chaharmahal and Bakhtiari province had the highest mean elevations, whereas Golestan province had the lowest (Figure 1). The highest and lowest NDVI values were observed in Mazandaran and Kerman provinces, respectively. Figure 1 presents the distribution of BPL rates in the studied provinces, along with the PCR positive and the mean DEM and NDVI values for each province (Figures 2 and 3). No significant correlations were found between prevalence of BPL in different provinces and elevation (*r* = −0.115, *p* = 0.8) and NDVI (*r* = −269, *p* = 0.56) though negative *r* means inverse relation between elevation and vegetation with BPL rate.

Although no difference was found between *Lophomonas* prevalence and provinces, a trend was found between *Lophomonas* prevalence and geographical latitudes. This was found by comparison *Lophomonas* positive rate and refereeing patients between north (Mazandaran, Golestan, Ardabil, Tehran, and Khorasan Razavi) and south (Kerman and Charmahal and Bakhtiari) areas (*p* = 0.058).

The current study found no significant correlations between the BPL in different provinces and elevation (*r* = −0.115, *p* = 0.8) or NDVI (*r* = −0.269, *p* = 0.56). A negative correlation coefficient indicates an inverse relationship between elevation and vegetation with BPL. Although there was no difference in *Lophomonas* prevalence among provinces, a trend was observed between *Lophomonas* prevalence and geographical latitudes. A comparison of *Lophomonas* positive rates and referring patients between north (Mazandaran, Golestan, Ardabil, Tehran, and Khorasan Razavi) and south (Kerman and Chaharmahal and Bakhtiari) areas showed a trending difference (*p* = 0.058).

## 4. Discussion

The current study is the first to examine environmental factors affecting the prevalence of BPL, and it also includes the largest sample size to date. We found that 22.7% of investigated patients were positive for BPL using a molecular approach. The highest infection rate was observed in southern Iran, whereas the lowest was seen in northeastern Iran. We did not observe any significant differences between PCR-positive results and the living area (province) of suspected patients, NDVI, or DEM, although we noted a trend between geographical latitude and *Lophomonas* rate. Furthermore, we found a significant difference in the prevalence of *Lophomonas* infection among different age groups.

The standard approach to diagnosing BPL involves microscopic examination of BAL, tracheal aspiration, sputum, and nasal secretions [23]. However, distinguishing this muliflagellated cell from pulmonary epithelial cells can be challenging [24], and it depends on the laboratory staff's skills and experience as well as parasite load. The current study found a high prevalence rate of BPL in Iran. In Panama, microscopic examination of BAL specimens showed that 35% of individuals with pulmonary problems had BPL infection [25]. The prevalence of BPL can depend on various factors, including the genetics and lifestyle of the at-risk population, air quality, the population of invertebrate hosts, and environmental factors.

Elevation and vegetation are two of the most crucial environmental factors that can affect parasitic diseases and their transmission by impacting rainfall, humidity, human, and insect habitats. In our study, we found that the elevation and NDVI varied from 491 to 2292 m and 0.09–0.59, respectively, among the studied areas, but no correlation was found between BPL prevalence and these factors. To date, there have not been any studies conducted to investigate the effect of environmental factors on BPL and *Lophomonas* epidemiology, although some reports have suggested potential effects. Ding and Shen demonstrated that BPL is a disease related to filthy and muggy environments due to the role of cockroaches in transmission [7]. Additionally, the presence of adequate humidity, oxygen, and suitable temperature is presumed to affect the excystation of *Lophomonas* [26]. Other studies have hypothesized that the patient's environment may contain moisture, dust, and cockroaches [18].

Although we did not find any association between environmental factors or provincial distribution and BPL prevalence, a comparison between provinces located at higher (North) and lower geographical latitudes (South) showed a significant difference in BPL prevalence. There is an association between sunlight severity and geographical latitude, where areas located at higher latitudes receive less sun exposure. On the other hand, southern provinces of Iran have warmer conditions and fewer cloudy days compared with the northern provinces, resulting in more indoor time and activity for people in the southern provinces. Spending more time indoors may lead to greater exposure to domestic insects like cockroaches and termites, as well as dust infected with these insects. For the first time in Iran, *Lophomonas* spp., was detected within the guts of German cockroaches (*Blattella germanica*) trapped in hospitals. The researchers stated that the presence of cockroaches in hospitals may pose a potential risk to patients and healthcare personnel [27].

A study in China hypothesized that reports of BPL cases from south of the Yangtze River may be related to the warm climate in this area [28]. However, differences in BPL distribution across latitudes may be due to other factors, such as genetic and ethnic differences in human populations or the effects of human movement on diseases and parasite genetics in different areas, as has been reported for the association of nomads as an immigrant population with some parasitic diseases in Iran [29]. Future studies should evaluate all of these hypotheses. Approximately 49% of confirmed patients were more than 60 years old, and up to 80% of cases were 41 years old or older, with an average age of 56 years old. A study in Khorasan Razavi province reported suspected cases ranging from 17 to 80 years old, with an average age of 43 years among four confirmed patients [18]. Talebian et al. found that the average age was 59 years old, with a range of 17–84 years old, although the prevalence of lophomoniasis was higher in children [27]. In a study in Peru, the age range of seven confirmed patients was from 9 to 95 years old, and only two patients were children [30].

In Turkey, nine confirmed cases of immunocompromised lophomoniasis were reported in patients aged between 48 and 78 years old [8]. While the disease seems to affect individuals over 40 years of age in most studies, a prevalence rate of 40% was found among children under 16 years old in a study conducted in Iran [9], and a mean age of 7 years was reported for 53 infected patients from a pediatric hospital in China [7]. Additionally, there have been some case reports of pediatric lophomoniasis, mostly in immunocompromised conditions [17]. Although some earlier studies concluded that the disease is more prevalent in children, it appears that the disease has a wide range of ages, with more cases occurring in adulthood. This may be related to higher exposure of adults to BPL risk factors, including dust in the home and workplace or a higher rate of pulmonary problems, especially chronic diseases in adulthood and elderly age.

In the current study, 68% of the cases were male. Martinez-Girón and van Woerden, in a review of 61 published cases, showed that 70.9% of patients were male. Virgilo Failoc-Rojas et al. in Peru and Ding and Shen in China reported that 60% and 56% of patients, respectively, were male [4, 7, 31]. Additionally, most case reports included males [31–33]. However, in rare studies, females were found to be the dominant gender. Ghafarian et al. reported that 57% of pediatric cases in northeast Iran were female, whereas 52.5% and 42.9% of adult and child cases, respectively, in that region were female [9].

All of the above-mentioned studies were descriptive, however, based on the only registry-based study conducted by Fakhar et al., which investigated that the relationship between gender and *Lophomonas* infection found a significant higher prevalence of the infection among male (60%) rather than female (40%) in patients from Mazandaran northern Iran [34]. However, in our current study encompassing different provinces of Iran, we did not find any association between gender and infection [28]. Therefore, it seems that male infection has been reported more frequently, but statistical associations between gender and infection should be further investigated in future studies.

## 5. Conclusion

As previously stated, the current registry-based study includes the greatest number of BPL cases in the world to date, which was also confirmed by a DNA-based molecular approach. Additionally, it is the first GIS-based study to investigate environmental factors associated with the prevalence of BPL. The prevalence of BPL among pulmonary patients in Iran was found to be 22.7%, and this was associated with geographical latitude and age, with most patients being over 40 years old. This may be related to higher exposure to dust and/or chronic pulmonary problems in older adults. The mean rate of BPL was higher in lower latitude provinces where the warmer climate and sunnier days can confine individual activities indoors, leading to greater contact with domestic insects and related infected dust.

However, no significant association was found between elevation, vegetation, or gender and the disease. Future studies could investigate the effect of environmental factors on the *Lophomonas* cycle in more confined areas with more precise data regarding patients' residential locations.

## Figures and Tables

**Table 1:** Distribution of BPLs according to age, gender, and provincial origin based on the PCR results.

Variables	PCR positive	PCR negative	*p*-Value
Age (years)			
1–10	3 (1.7)	41 (7.4)	0.027
11–20	6 (3.4)	16 (2.9)	
21–40	26 (14.5)	89 (16)	
41–60	57 (31.8)	193 (34.7)	
>61	87 (48.6)	217 (39)	
Total	179 (100)	556 (100)	—
Gender			
Female	83 (38.1)	282 (38.1)	0.993
Male	135 (61.9)	458 (61.9)	
Total	218 (100)	740 (100)	—
Provinces (no. of examined)			
Mazandaran (625)	137 (21.9)	488 (78.1)	**—**
Golestan (52)	11 (21.2)	41 (79.8)	
Ardabil (10)	3 (30)	7 (70)	
Chaharmahal-Bakhtiari (133)	36 (27.1)	97 (72.9)	
Tehran (78)	20 (25.6)	58 (74.4)	
Khorasan Razavi (49)	5 (10.2)	44 (89.8)	
Kerman (13)	6 (46.1)	71 (53.9)	
Total	218 (100)	740 (100)	958 (100)

## Data Availability

Data supporting this research article are available from the corresponding author or first author on reasonable request.
